# Supplementary material for: Non-volatile 2D MoS2/black phosphorus heterojunction photodiodes in the near- to mid-infrared region
Source: Nat Commun. 2024 Jul 17;15:6015. doi: 10.1038/s41467-024-50353-6 (PMC11255212; doi:10.1038/s41467-024-50353-6)
Supplement: Supplementary file 1 — Supplementary Information [file 41467_2024_50353_MOESM1_ESM.pdf]

Supplementary information

Non-volatile 2D MoS_2/black phosphorus heterojunction photodiodes in the near- to mid-infrared region

Yuyan Zhu[1,§], Yang Wang[1,2,3,4,§*], Xingchen Pang[1], Yongbo Jiang[1], Xiaoxian Liu[1], Qing Li[2,5], Zhen Wang[2], Chunsen Liu[1,6], Weida Hu[2,5*], Peng Zhou[1,3,4,6*]

[1]State Key Laboratory of ASIC and System, School of Microelectronics, Fudan University, Shanghai 200433, China

[2]State Key Laboratory of Infrared Physics, Shanghai Institute of Technical Physics, Chinese Academy of Sciences, Shanghai 200083, China

[3]Shaoxin Laboratory, Shaoxing 312000, China

[4]Shanghai Frontiers Science Research Base of Intelligent Optoelectronics and Perception, Institute of Optoelectronics, Fudan University, Shanghai 200433, China

[5]Hangzhou Institute for Advanced Study, University of Chinese Academy of Sciences, Hangzhou 310024, China

[6]State Key Laboratory of Integrated Chip and System, Frontier Institute of Chip and System, Fudan University, Shanghai 200433, China

[§]These authors contributed equally: Yuyan Zhu, Yang Wang

*Email: yang_wang@fudan.edu.cn; wdhu@mail.sitp.ac.cn; pengzhou@fudan.edu.cn

Table of contents

Supplementary Note 1. The fabrication process..3

Supplementary Note 2. The characterization of the PMC device materials..4

Supplementary Note 3. Validation test of memory and mid-wave infrared detection structure...8

Supplementary Note 4. Electrical characteristics and laser response characteristics of the PMC device...13

Supplementary Note 5. Black radiation response of the PMC device...30

Supplementary Note 6. Image processing application of the PMC device...35

Supplementary Note 1. The fabrication process

We chose SiO_2/Si (300 nm SiO_2 grown on p-doped Si substrates) as the substrate, initially cleaning it with acetone and ethylene glycol for a smooth surface. Subsequently, we transferred the two-dimensional materials on a fixed-point transfer platform. All of the 2D materials (BP, MoS_2, *h*-BN and graphene) flakes were mechanically exfoliated using polydimethylsiloxane (PDMS) from bulk crystal (from HQ Graphene). Through dry transfer technology, the graphene, *h*-BN, MoS_2 and BP were firstly transferred to the substrate in turn.

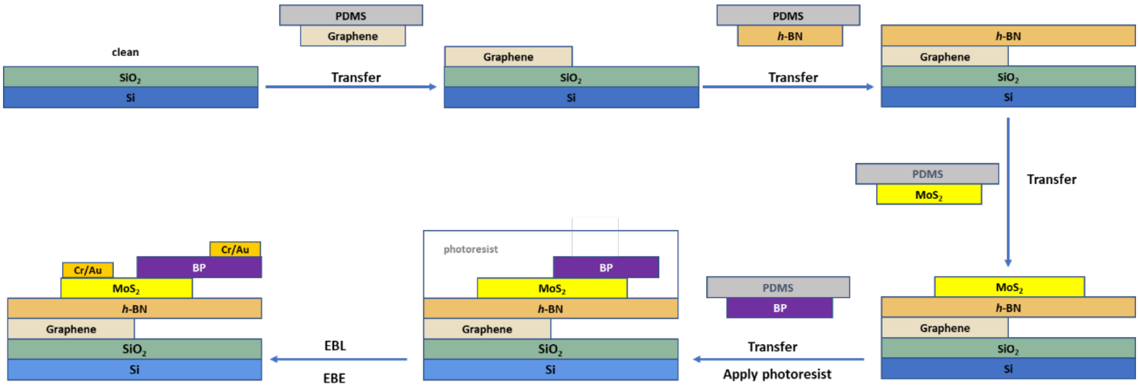

Supplementary Fig. 1 | Schematic diagram of the fabrication of the device.

Supplementary Note 2. The characterization of the PMC device materials

Supplementary Fig 2 shows the optical images of the device and atomic microscope images. The typical thicknesses of materials (BP/MoS_2/*h*-BN/graphene) of PMC functional devices are measured at the same time.

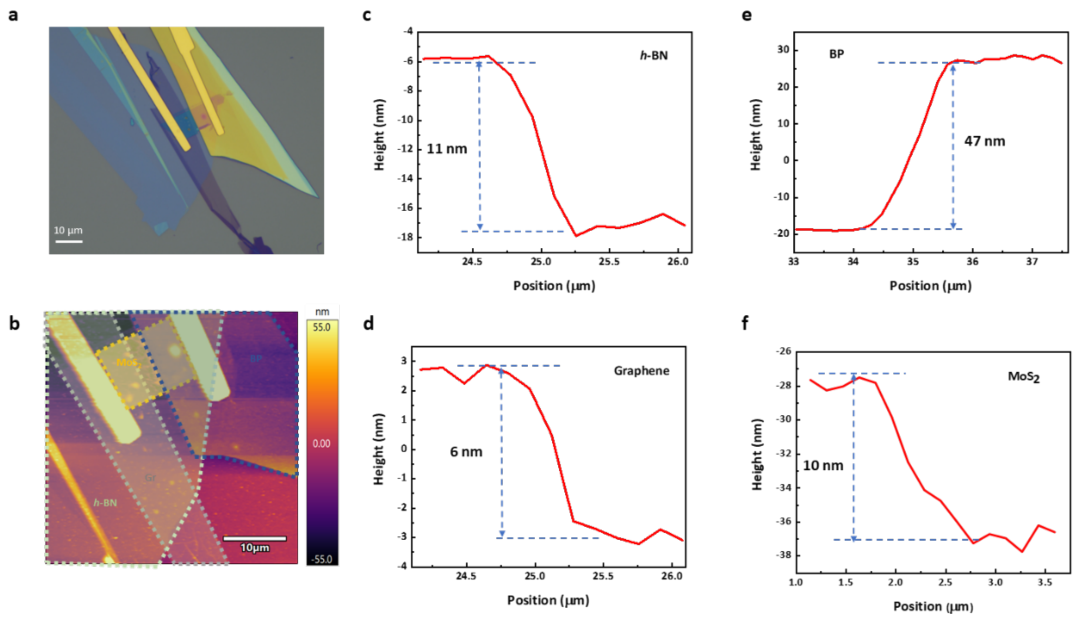

Supplementary Fig. 2 | The typical thicknesses of materials. a, Top view of the optical microscope photograph of the PMC device on a low-resistance silicon substrate with a 300-nm-thick SiO_2 (scale: 10 μm). **b,** Atomic microscope images of PMC device. The stacking order of the heterostructures is marked from top to bottom by blue, yellow, green and purple dashed lines representing BP, MoS_2, *h*-BN and graphene, respectively. The scale bar is 10 μm. **c, d, e, f,** Typical thicknesses: *h*-BN (11 nm), graphene (6 nm), BP (47 nm), MoS_2 (10 nm), respectively.

Supplementary Fig 3 shows the structure and energy dispersive X-ray spectroscopy mapping (scales are 5 nm). EDS point, line and surface analysis data were obtained using the Super X FEI System in STEM mode.

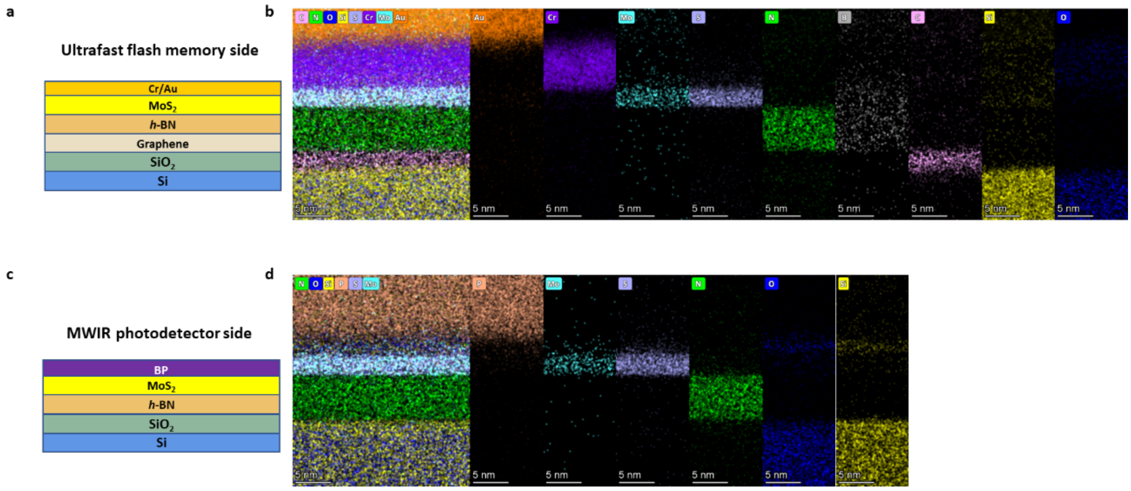

Supplementary Fig. 3 | Energy dispersive X-ray spectroscopy mapping. a, c, Vertical layered structure diagram for ultrafast flash memory on the left of the PMC device and MWIR detection on the right. **b, d,** Corresponding energy dispersive X-ray spectroscopy mapping (scales are 5 nm).

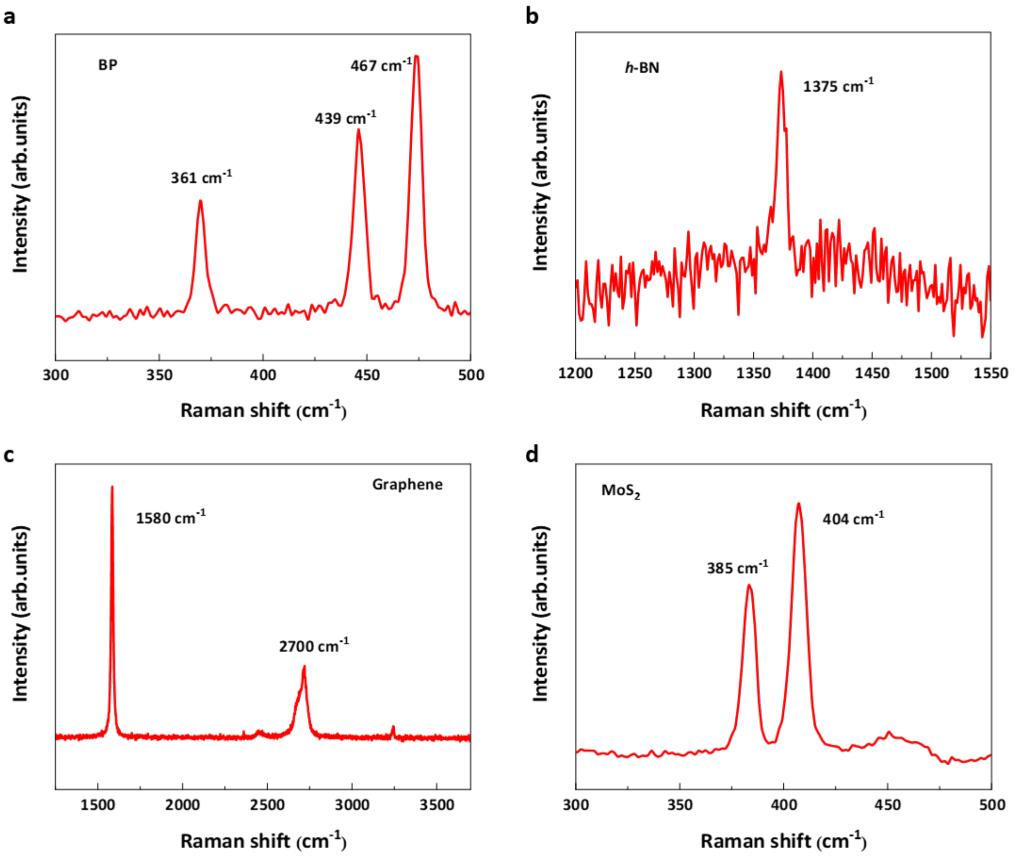

Supplementary Fig. 4 | Raman spectra of the PMC device's materials. a, Typical Raman shift of BP at 361 cm^{-1}, 439 cm^{-1} and 467 cm^{-1}. **b,** Typical Raman shift of *h*-BN at 1375 cm^{-1}. **c,** Typical Raman shift of graphene at 1580 cm^{-1} and 2700 cm^{-1}. **d,** Typical Raman shift of MoS_2 at 358 cm^{-1} and 404 cm^{-1}.

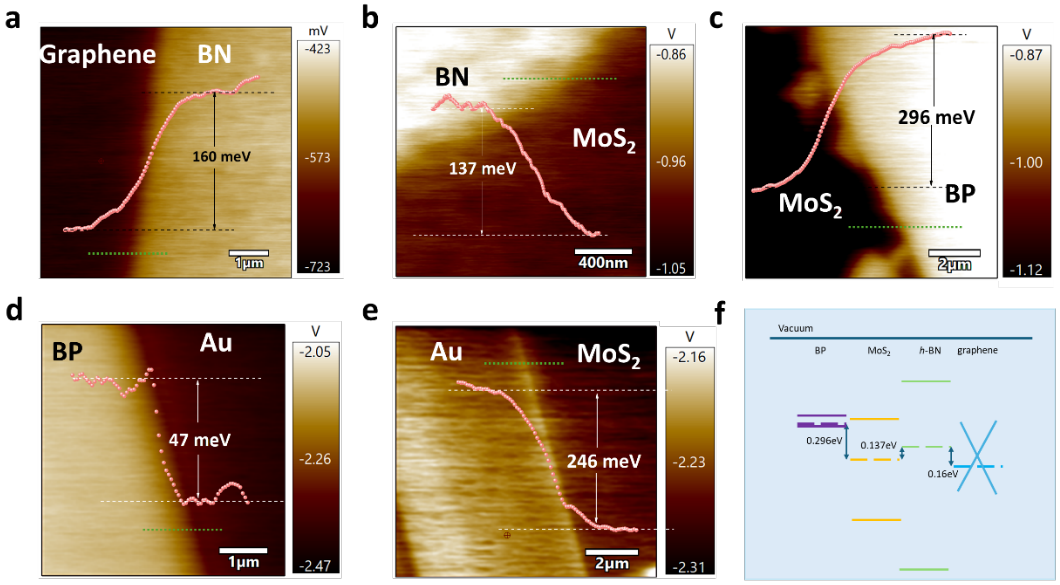

Supplementary Fig. 5 | Kelvin Probe Force Microscope (KPFM) for material interface barriers. a-e, interface barriers between (graphene, BN) 160 meV, (BN, MoS_2) 137 meV, (BP, MoS_2) 296 meV, (BP, Au) 47 meV and (MoS_2, Au) 246 meV, respectively. **f,** Alignment of energy bands before contact.

Supplementary Note 3. Validation test of memory and mid-wave infrared detection structure.

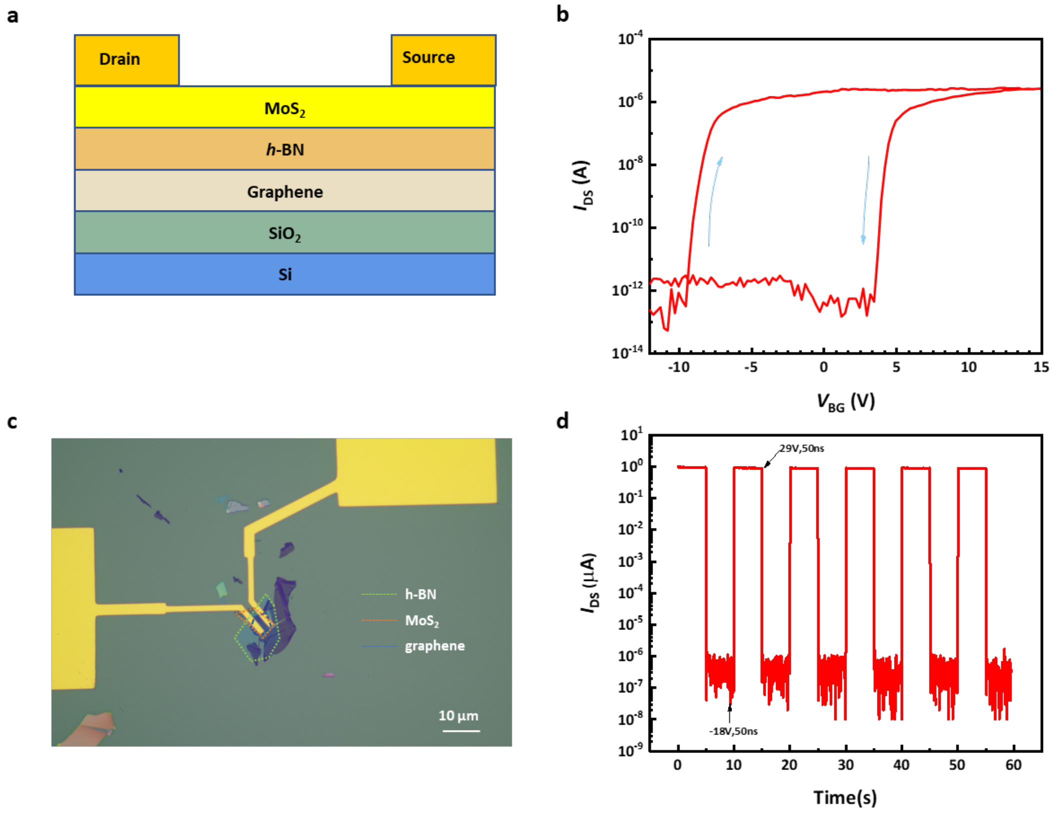

Supplementary Fig. 6 | The performance of the MoS_2/*h*-BN/graphene flash memory. a,c, Flash memory structure and the top view of the optical microscope photograph of the device**. b,** Transfer characteristics of the device controlled by the back gate. The shift of the threshold voltage leads to the generation of the memory window at V_{DS}= 1 V when V_{BG} sweeps from -12 V to 15 V. **d,** Variation of channel current values after the writing (V_{BG}, pulse= +29 V for 50 ns) and erasing (V_{BG}, pulse= −18 V, 50 ns) operations. A large memory current state-1/state-0 ratio was observed which exceeded 10^6, reading at V_{DS}= 1 V and V_{BG}= 0 V.

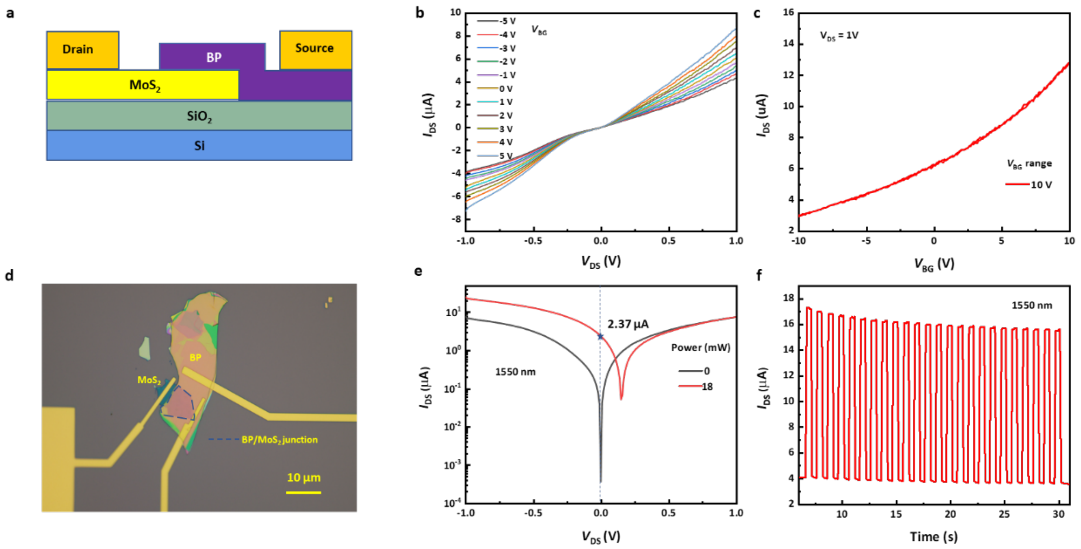

Supplementary Fig. 7 | Infrared detection of BP/MoS_2 heterojunction. a, BP/MoS_2 heterojunction structure controlled by a back gate. **b,** Output characteristic curve. The I_{DS} changes from 4 μA to 8 μA at V_{DS} = 1 V when V_{BG} sweeping from -5 V to 5 V. **c,** Transfer characteristics of the device controlled by the back gate. Increasing the V_{BG} sweeping range to 10 V, but the transistor remains ineffective in being turned off. **d,** Top view of the optical microscope photograph of BP/MoS_2 heterojunction. The heterojunction region is marked by the blue dashed line. **e,** I_{DS}-V_{DS} curve of the BP/MoS_2 photodetector in the dark and under 1550 nm illumination, and the photocurrent reaches 2.37 μA at zero bias (1550 nm, 16 μW/μm^2). **f,** Continuous period photo-response with the dark current displayed under 1500 nm switching conditions at V_{DS}= -1 V.

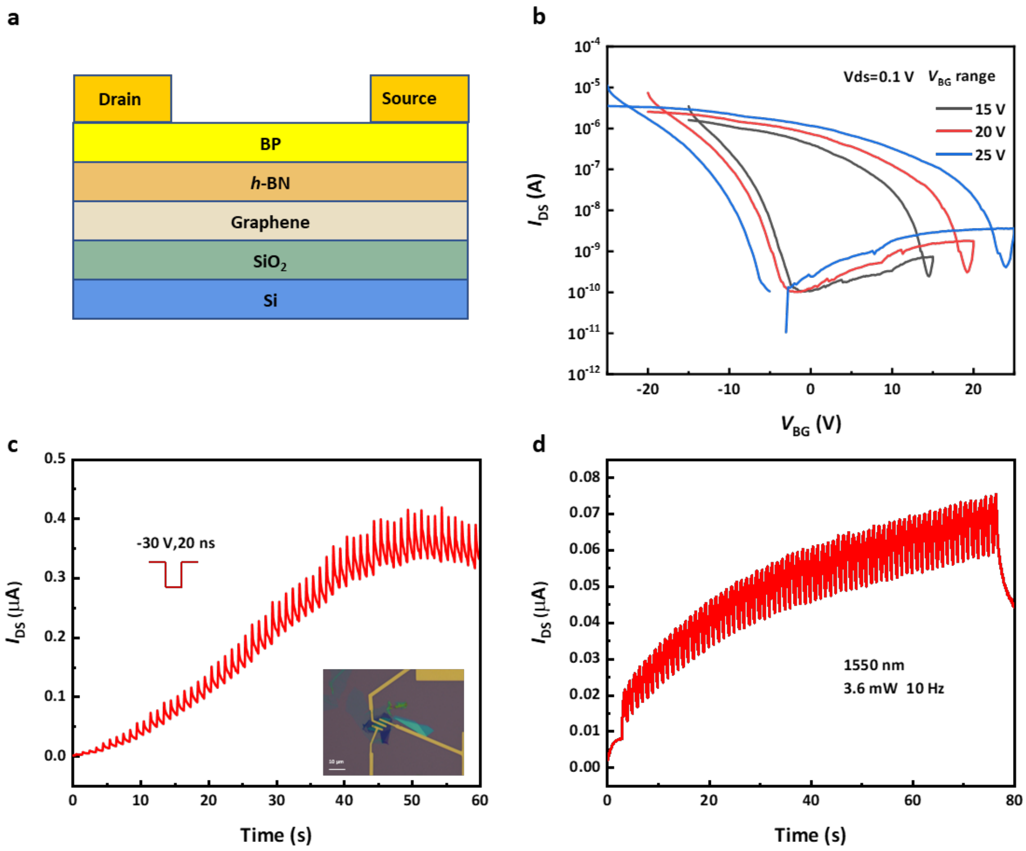

Supplementary Fig. 8 | Electrical characteristics and infrared laser regulation of BP/*h*-BN/graphene flash memory. a, BP/*h*-BN/graphene flash memory structure controlled by a back gate. **b,** Transfer characteristic curve with memory windows. The dynamic range of electrical regulation is over 10^4. **c,** Applying an electrical pulse set of (V_{BG}, pulse= -30 V for 20 ns, 1 Hz) regulates the conductance state from 1 pA to 350 nA. Inset: top view of the optical microscope photograph of BP flash memory device. The scale bar is 10 μm. **d,** The conductance state can be regulated by the 1550 nm laser pulse. A maximum photoresponsivity of 0.0125 A/W is obtained at 2.4 mW mm^{-2}. The BP flash memory device shows characteristics of a volatile synapse. Challenges arise in achieving both electrical modulation and optical absorption due to the need for thin materials for electrical modulation and thick materials for optical absorption. Therefore, this necessitates using thin materials for conductive state changes and thick materials for optical absorption.

MoS_2 and h-BN are all thicker, with typical values of around 20 nm. This thickness configuration results in a PMC device with a small range of conductance state regulation and no memory window in **Supplementary Fig. 9c**. Typical values of around 10 nm MoS_2 and 20 nm *h*-BN. This thickness configuration results in a PMC device with a large range of conductance state regulation (ON/OFF ratio 10^6) and no memory window in **Supplementary Fig. 9f.** Typical values of around 20 nm MoS_2 and 10 nm *h*-BN. This thickness configuration results in a PMC device with a small range of conductance state regulation with a considerable memory window in **Supplementary Fig. 9i.**

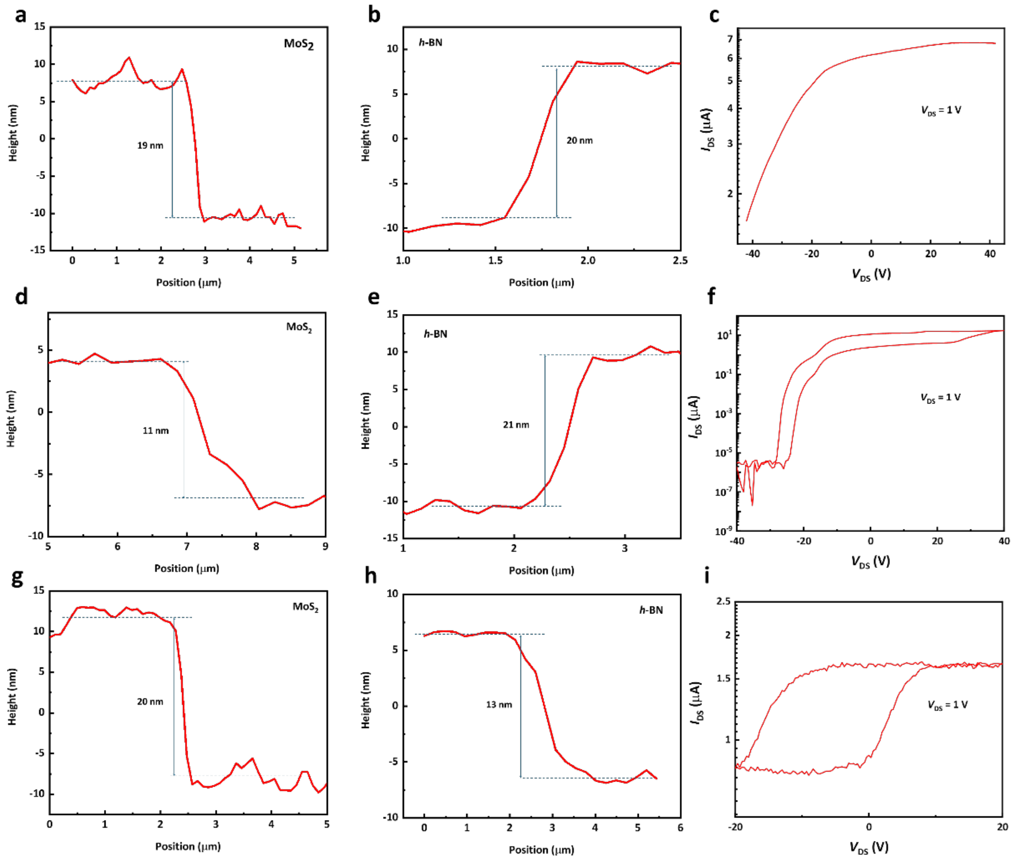

Supplementary Fig. 9 | The device performance of different thicknesses MoS_2 and *h*-BN. a, b, the thickness of MoS_2 (19 nm) and *h*-BN (20 nm). **c,** I_{DS}-V_{BG} curve of the PMC device with V_{DS} = 1 V. **d, e,** the thickness of MoS_2 (11 nm) and *h*-BN (21 nm). **f,** I_{DS}-V_{BG} curve of the PMC device with V_{DS} = 1 V. **g, h,** the thickness of MoS_2 (20 nm) and *h*-BN (13 nm). **i,** I_{DS}-V_{BG} curve of the PMC device with V_{DS} = 1 V.

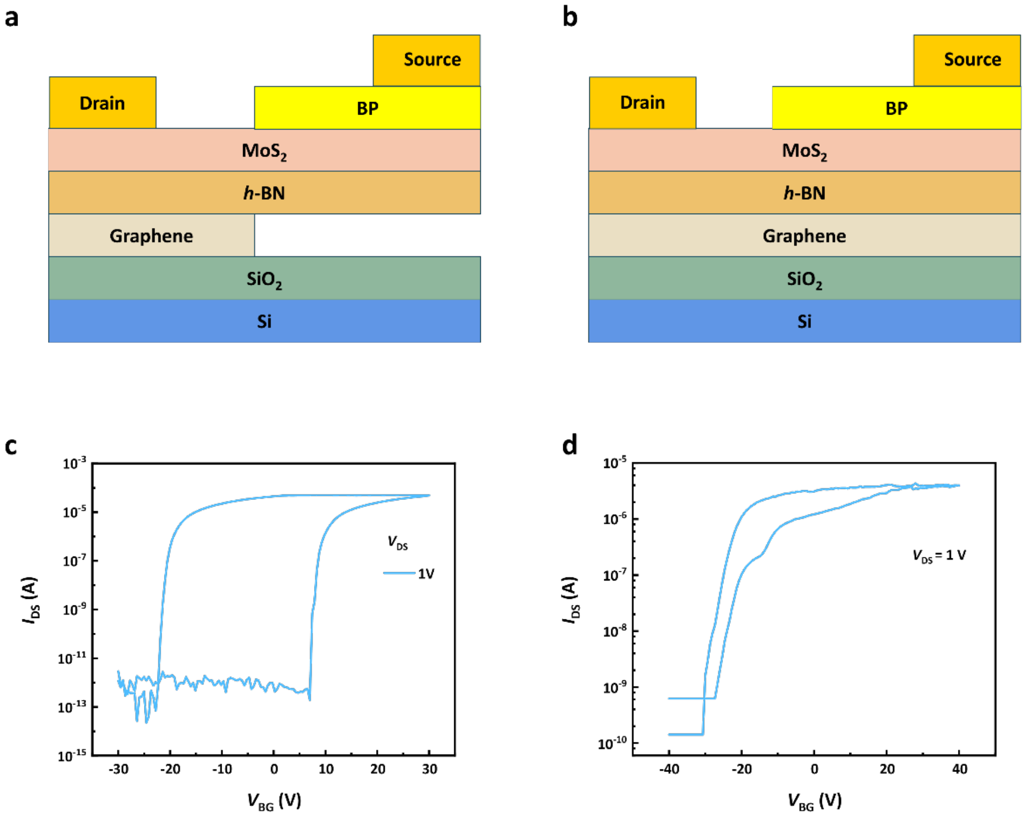

Supplementary Fig. 10 | Memory performance comparison between the semi-floating gate structure and directly stacked structure. a, Device structure diagram of the semi-floating gate. **b,** Diagram of the directly stacked device structure. **c,** Device memory performance of the semi-floating gate. **d,** Memory performance of directly stacked device structure.

Supplementary Note 4. Electrical characteristics and laser response characteristics of the PMC device.

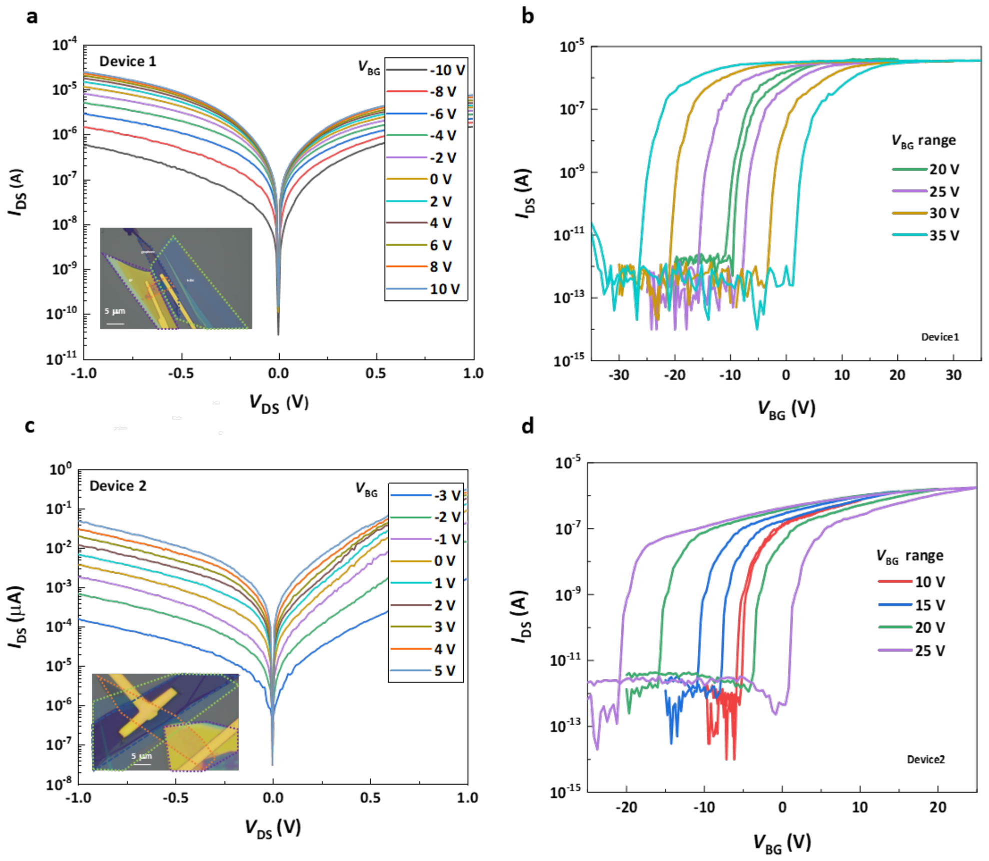

Supplementary Fig. 11 | Electrical characteristics of the PMC device. a, b, Output characteristic curve and transfer characteristic curve of device 1, respectively. The memory window caused by the shift of the threshold voltage reaches 30 V at V_{DS} = 1 V when V_{BG} sweeps from -35 V to 35 V and owns a dynamic range of more than 10^6. **c, d,** Output characteristic curve and transfer characteristic curve of device 2, respectively. The memory window reaches 20 V at V_{DS} = 1 V when V_{BG} sweeps from -25 V to 25 V and owns a dynamic range of more than 10^5 (slightly lower than device 1 caused by a thicker *h*-BN layer). Insets **a** and **c** show the top view of the optical microscope photographs of the PMC device. The scale bar is 5 μm.

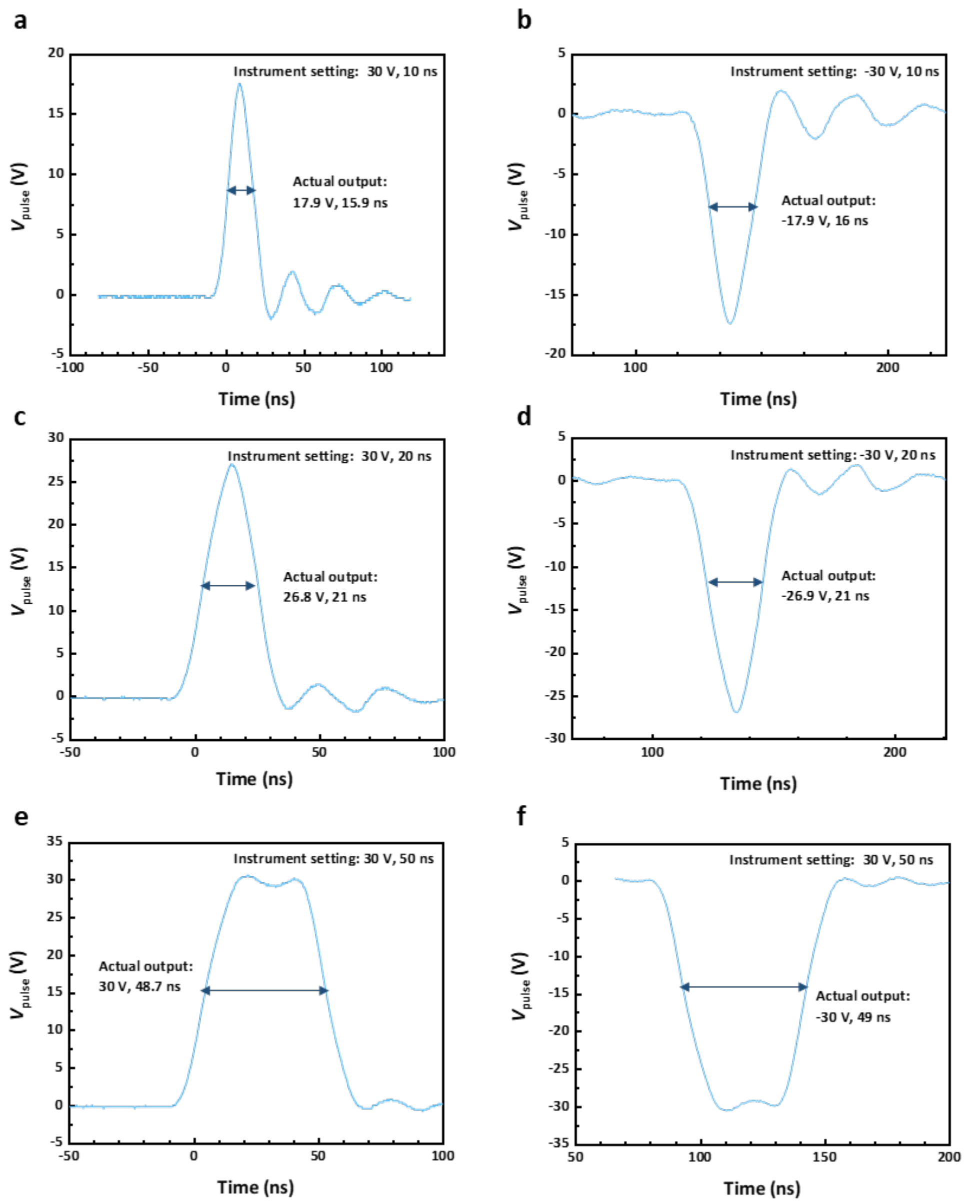

Supplementary Fig. 12 | The voltage pulses after the transmission process. a, c, e, Pulses with 30 V voltage amplitude and pulse width with 10 ns, 20 ns and 50 ns generated by semiconductor analyzer (B1500 SPGU) respectively and captured by oscilloscope. The corresponding effective duration of the pulse (FWHM, full width at half maximum) are 15.9 ns, 21 ns and 48.7 ns, respectively. **b, d, f,** Pulses with -30 V voltage amplitude and pulse width with 10 ns, 20 ns and 50 ns, generated by B1500, respectively. The corresponding FWHM are 16 ns, 21 ns and 49 ns, respectively.

Pulse modulation effect

The electric field in h-BN and MoS_2 layers is around 14 MV cm^{-1} when a 30 V or -30 V voltage is applied, which is much larger than the 7.43 MV cm^{-1} that can induce the FN tunneling effect in the Cr/h-BN/graphene heterostructure. So, the FN tunneling current of carriers should be large in the PMC device and play an important role in the short writing time. Continuing to increase the gate voltage theoretically leads to higher tunnelling currents and program time can be as low as 1 ns[1]. However, the minimum pulse width of the instrument (B1500 SPGU) limits further exploration (**Supplementary Fig. 12**).

Different pulse settings tend to have a direct effect on the electrical dynamic range of the device, the number of states stored in the device and the average state interval. Here we tested the modulation of some pulses in **Supplementary Fig. 13**. By analyzing the test results, the following main conclusions were summarized in **Supplementary Table 1.** (1) For pulses of the same time width, increasing pulse amplitude results in a larger conductance modulation range and a smaller number of conductance states. (2) For pulses of the same amplitude, increasing pulse width leads to a larger conductance modulation range and a smaller number of conductance states. In the table, we quantitatively show the range of conductance states, the number of conductance states and the average conductance interval corresponding to the pulse modulation. These findings contribute to a comprehensive understanding of the impact of pulse parameters on device performance.

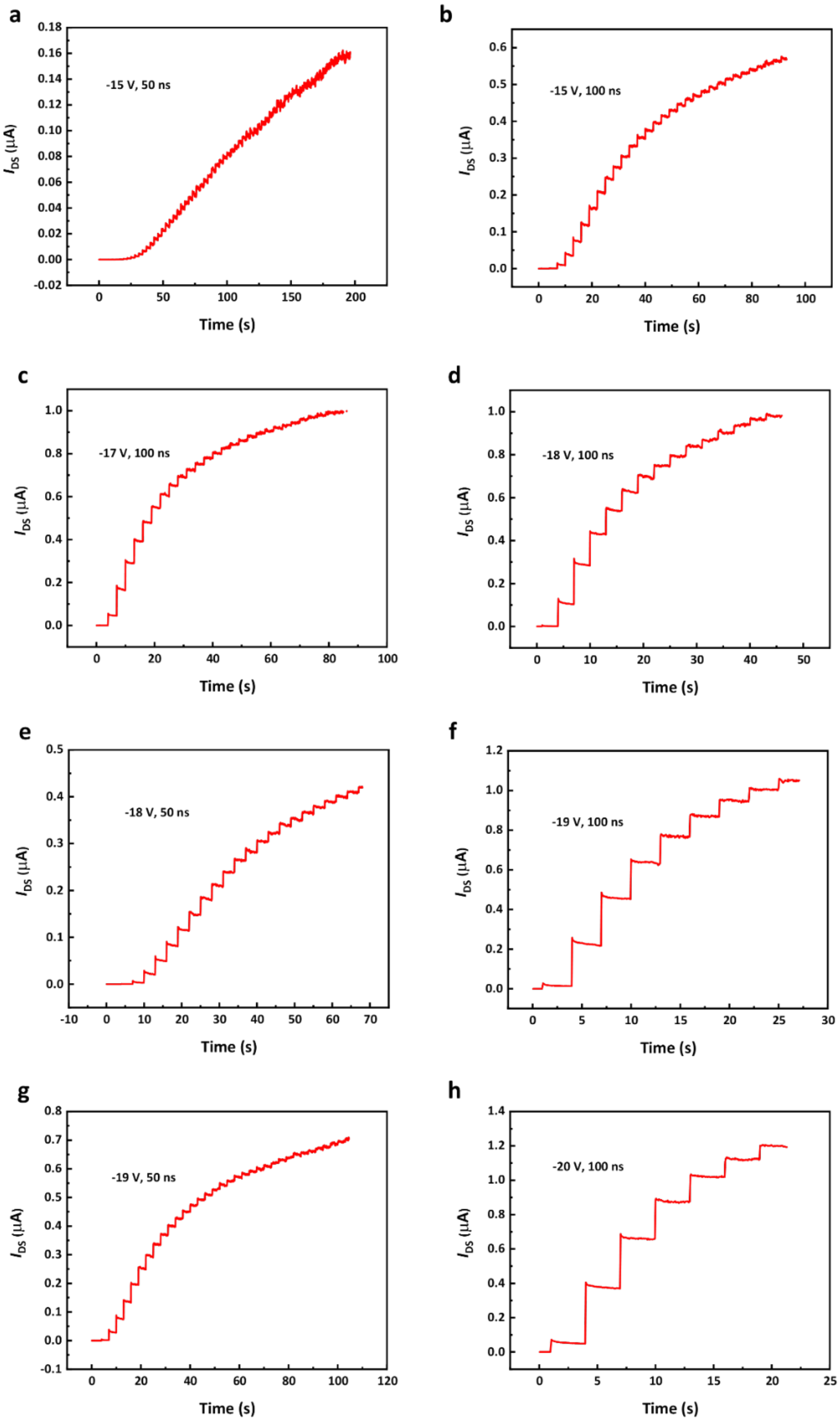

Supplementary Fig. 13 | Pulse modulation under various pulse sets. a-h, pulse modulation results under pulse sets (-15 V, 50 ns), (-15 V, 100 ns), (-17 V, 100 ns), (-18 V, 100 ns), (-18 V, 50 ns), (-19 V, 100 ns), (-19 V, 50 ns) and (-20 V, 100 ns), respectively.

Supplementary Table 1. Pulse modulation results.

V_{GS} and Pulse time	States number	Dynamic range (µA)	average conductance interval (µA)
-15 V, 50 ns	52 (5.7bits)	0.16	0.003
-15 V, 100 ns	30 (4.9bits)	0.56	0.0187
-17 V, 100 ns	27 (4.7bits)	1	0.037
-18 V, 100 ns	15 (3.9bits)	0.96	0.064
-18 V, 50 ns	22 (4.6bits)	0.4	0.0182
-19 V, 100 ns	10 (3.3bits)	1	0.1
-19 V, 50 ns	33 (5.0bits)	0.7	0.0212
-20 V, 100 ns	8 (3bits)	1.2	0.15

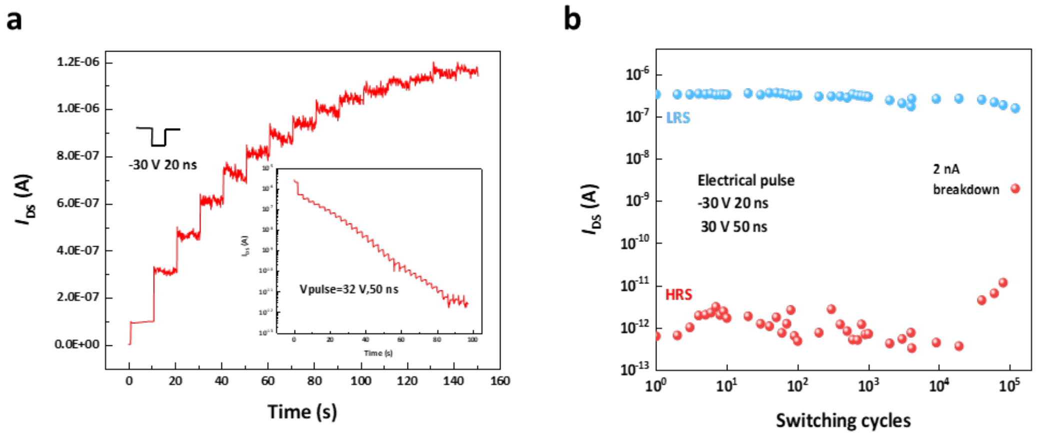

Supplementary Fig. 14 | Reconfigurable characteristics and endurance testing. a, reconfiguration features between 1 pA and 1 µA dynamic ranges (20 ns pulse width). **b,** Device endurance test. The -30 V/20 ns is used to program the device to high resistance state (HRS), and the 30 V/1s is used to program the device to a low resistance state (LRS). The channel current is read out at V_{BG} = 0 V, V_{DS} = 1 V. The PMC device maintains stable operation for up to 10^4 pulse cycles, with the low conductivity state failing at 2 nA after 10^5 pulse cycles.

Retention time mechanism

The loss of the conductance state is mainly due to the leakage of the captured charge by tunneling under weak fields, which is thus manifested by a change in the threshold voltage. Here we define the conductance state failure criterion as a 50% loss of captured charge, in other words, the time corresponding to the shift of the threshold voltage difference to 50% of the initial value is the conductance retention time. In the **Fig. 3a** of the previous manuscript, we presented the retention time of approximately 1000s. To further elucidate the retention performance, we conducted extensive measurements of the retention performance based on the shift of the threshold voltage, as depicted in **Supplementary Fig. 15a-c**. These results further clarify the methodology for determining the retention time of our device. Device data can be maintained for several years with this test method In **Supplementary Fig. 15d**, we also performed exponential fitting of I_{ds} against time using the function Asymptotical (y=a-b*cx) for both low resistance state and high resistance state (LRS and HRS). we make an exponential fitting of the I_{ds} against time. The exponential fit function is Asymptotical ($y=a-b*c^x$). LRS (a=1.56E-12, b=-7.37E-13, c=0.99794); HRS (a=1.02E-5, b=-3.28E-6, c=0.99939). From the results of the exponential fit, we estimate that the high and low resistance states of the device can be held for up to 10 years.

In consideration of the susceptibility of BP material to oxidation, we coated a layer of PMMA on the surface of the device to insulate it from moisture and oxygen. We show the original memory characteristics of PMC devices in **Supplementary Fig. 16.** As a comparison, we show the memory characteristics of PMC devices after 15 days in **Supplementary Fig. 17.** The results show that the PMMA coating can effectively reduce the effect of BP oxidation on the device performance.

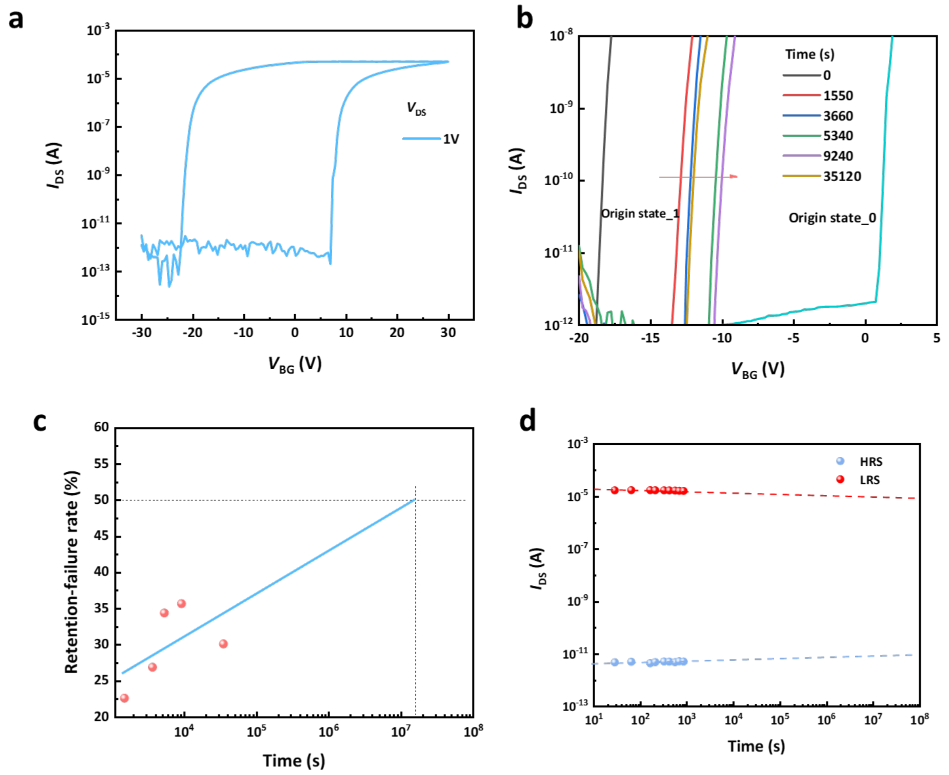

Supplementary Fig. 15 | Verification of non-volatile retention characteristics. a, I_{DS}-V_{BG} hysteresis curve of the PMC device with V_{DS} = 1V, **b,** Transfer characteristic curves of the PMC device (after a V_{BG} pulse= -30 V for 50 ns duration erasing operation) at a different time interval. The original state_0 was achieved by 30 V for 1 s pulse. **c,** Retention-failure rates for the device. **d**. Exponential fitting of the I_{DS} against time. From the results of the exponential fit, the high and low states of the device can be held for up to 10 years.

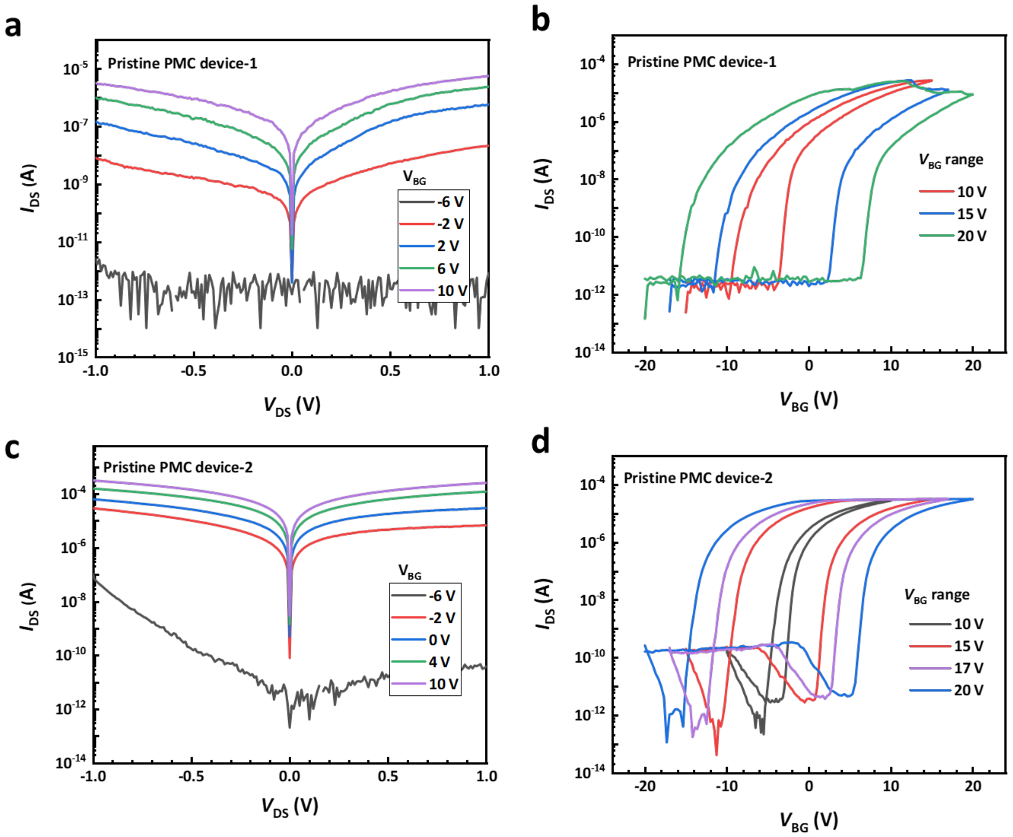

Supplementary Fig. 16 | Memory characteristics of pristine PMC devices. a, b, I_{DS}-V_{DS} curve of the PMC device-1 with different V_{BG}. I_{DS}-V_{BG} hysteresis curve of the PMC device-1 with V_{DS} = 1V. **c, d,** I_{DS}-V_{DS} curve of the PMC device-2 with different V_{BG}. I_{DS}-V_{BG} hysteresis curve of the PMC device-2 with V_{DS} = 1V.

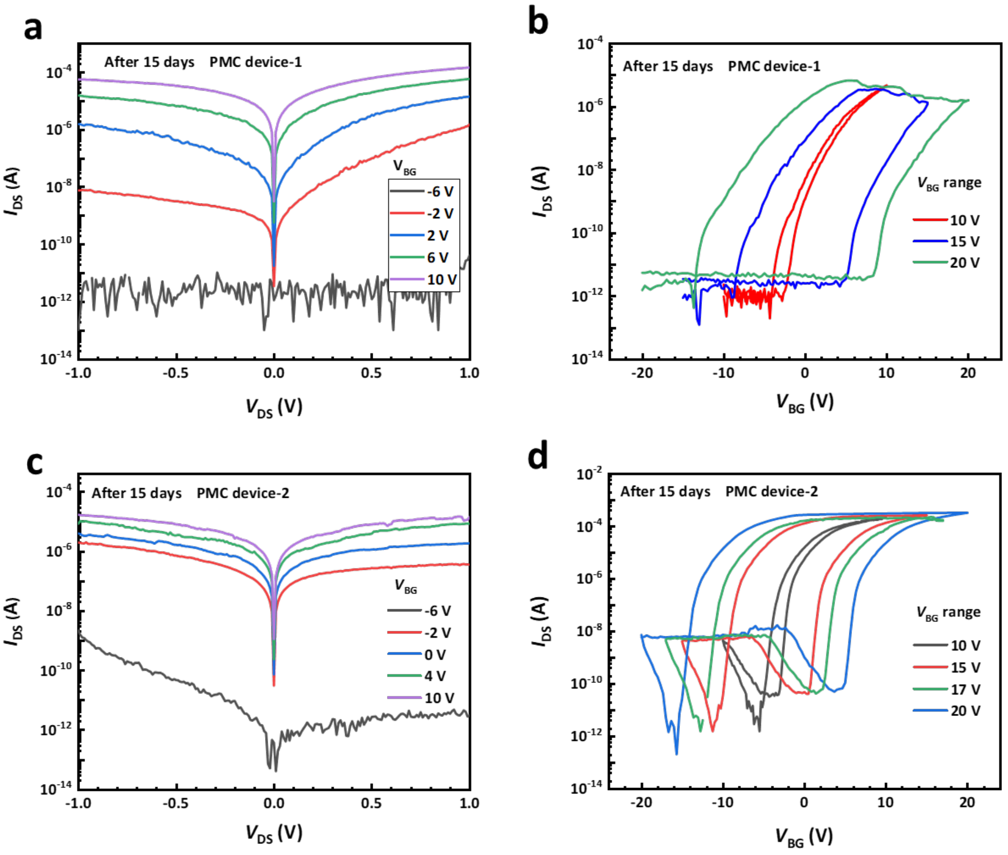

Supplementary Fig. 17 | Memory characteristics of PMC devices after 15 days. a, b, I_{DS}-V_{DS} curve of the PMC device-1 with different V_{BG}. I_{DS}-V_{BG} hysteresis curve of the PMC device-2 with V_{DS} = 1V. **c, d,** I_{DS}-V_{DS} curve of the PMC device-2 with different V_{BG}. I_{DS}-V_{BG} hysteresis curve of the PMC device-2 with V_{DS} = 1V.

The saturation current of electricity is equivalent to the container, the introduction of visible light (638 nm) and increase of the light intensity will not change the saturation current of the device However, it leads to an elevation in the device's cutoff current. Simultaneously, the memory window slightly expands, as more carriers enter the floating gate layer under the drive of light, making threshold regulation more pronounced.

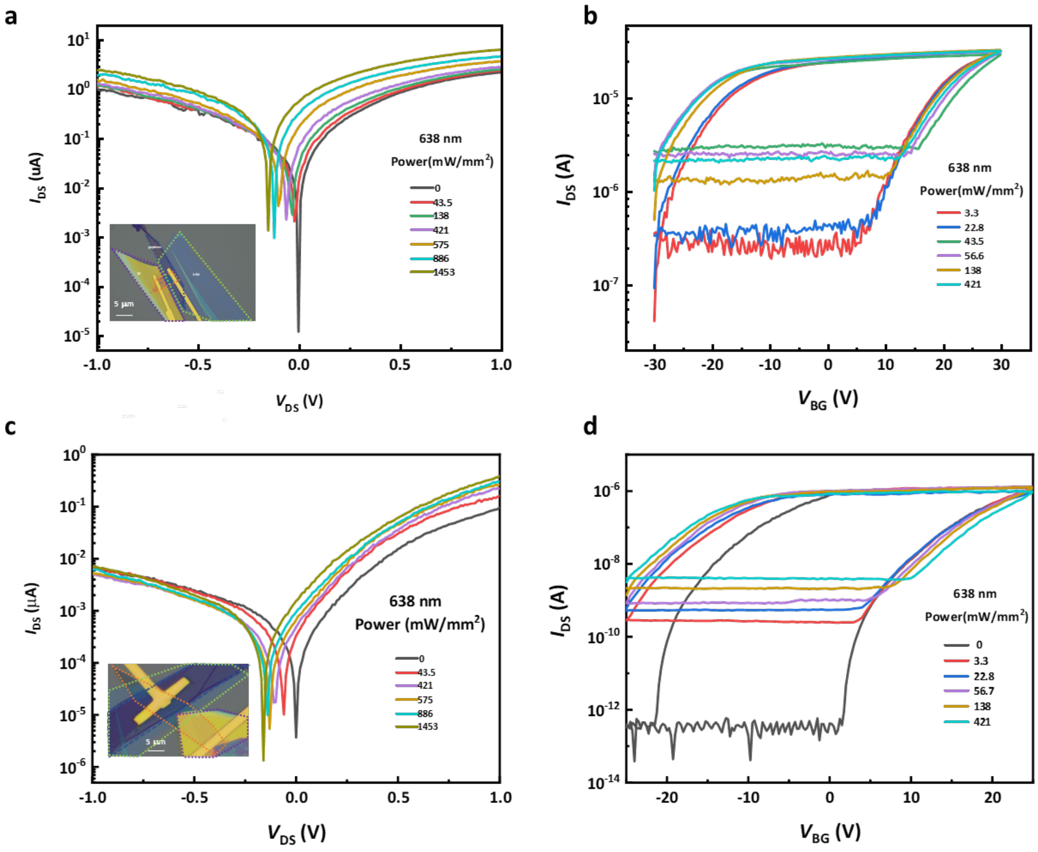

Supplementary Fig. 18 | The photoresponse of the PMC device at various power density under 638 nm laser stimulation. a, b, Output characteristic curve and transfer characteristic curve of device 1 at different power density, respectively. **c, d,** Output characteristic curve and transfer characteristic curve of device 2 at different power density, respectively. Insets of **a** and **c** show top view of the optical microscope photographs of PMC device. The scale bar is 5 μm.

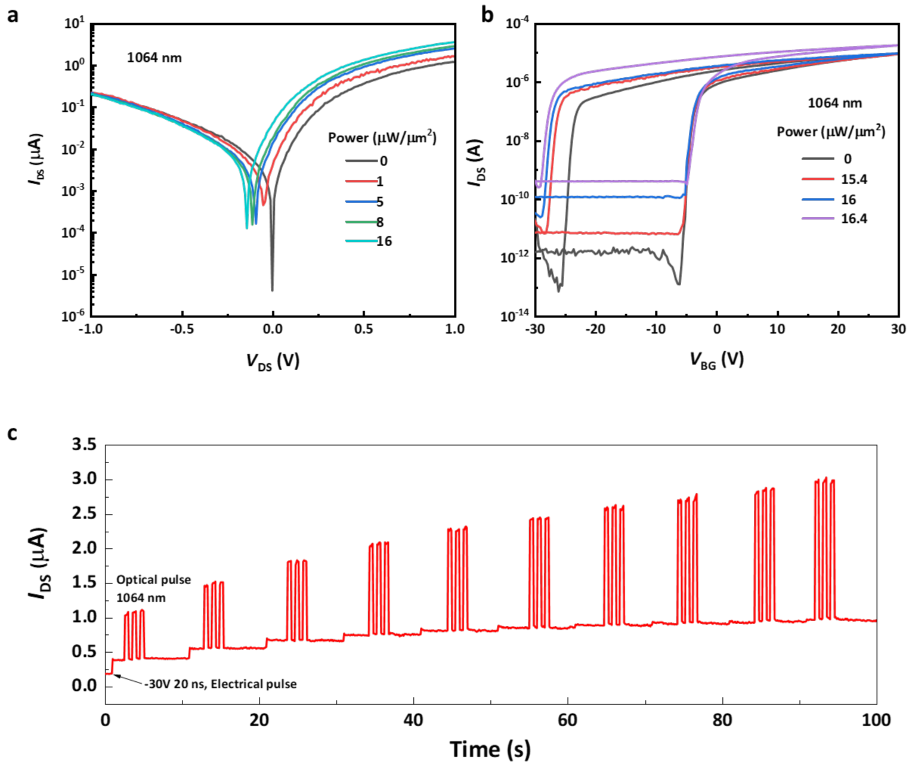

Supplementary Fig. 19 | The photoresponse of the PMC device under 1064 nm laser stimulation. a, b, Output characteristic curve and transfer characteristic curve of device 1 at different power density, respectively. **c,** Real time test for conductance configuration and the photoresponse at different conductance states. The laser pulse set (wavelength:1064 nm, power density:16 μW/μm^2, frequency:1 Hz). The electrical pulse set (amplitude: -30 V, pulse width: 20 ns, frequency:10 Hz).

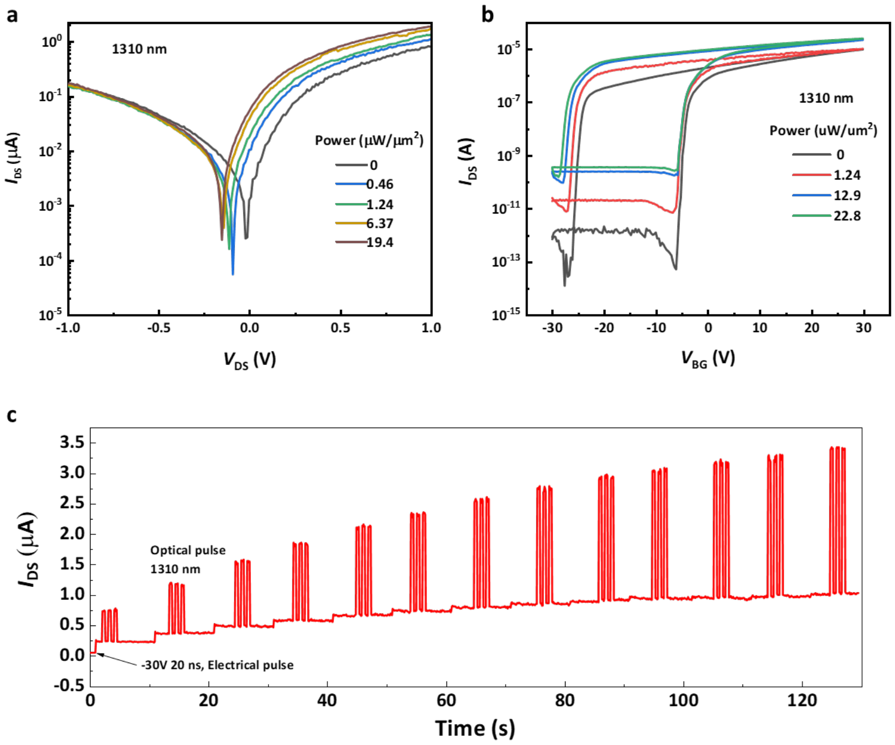

Supplementary Fig. 20 | The photoresponse of the PMC device under 1310 nm laser stimulation. a, b, Output characteristic curve and transfer characteristic curve of device 1 at different power density, respectively. **c,** Real time test for conductance configuration and the photo-response at different conductance states. The laser pulse set (wavelength:1064 nm, power density:20 $\mu W/\mu m^2$, frequency:1 Hz). The electrical pulse set (amplitude: -30 V, pulse width: 20 ns, frequency:10 Hz).

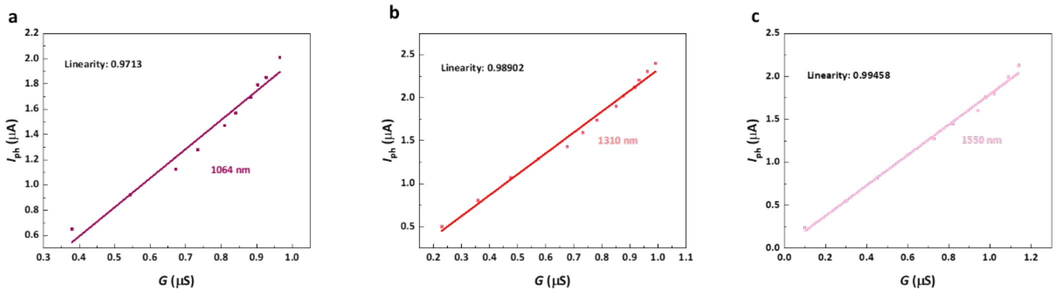

Supplementary Fig. 21 | The linear correspondence between the net photocurrent and the conductance at different wavelengths. a, The linear correspondence between the net photocurrent and the conductance at 1064 nm, R^2 = 0.9713. **b,** The linear correspondence between the net photocurrent and the conductance at 1310 nm, R^2 = 0.98902. **c**, The linear correspondence between the net photocurrent and the conductance at 1550 nm, R^2 = 0.99458

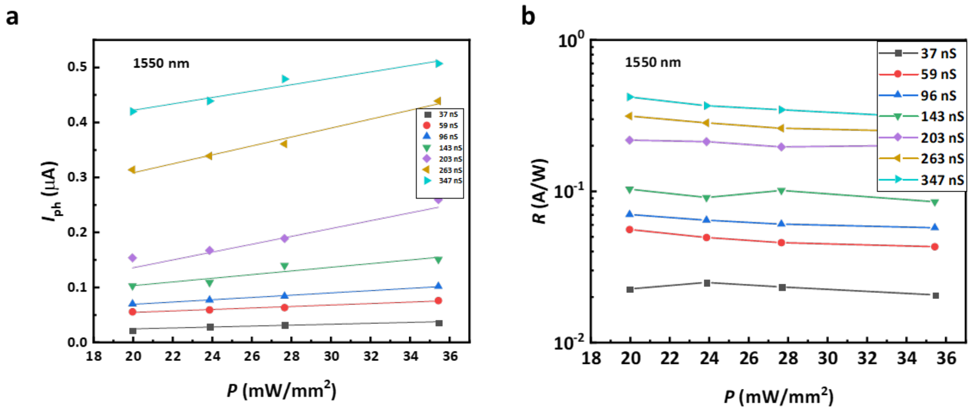

Supplementary Fig. 22 | Stable responsivity when the input power density changes. a, Linear relationship between net photocurrent and power density (1550 nm laser) at seven conductance (37 nS, 59 nS, 96 nS, 143 nS, 203 nS, 263 nS and 347 nS). **b,** Stable responsivity (calculated from the I_{ph} data of **a**) when the input power density changes.

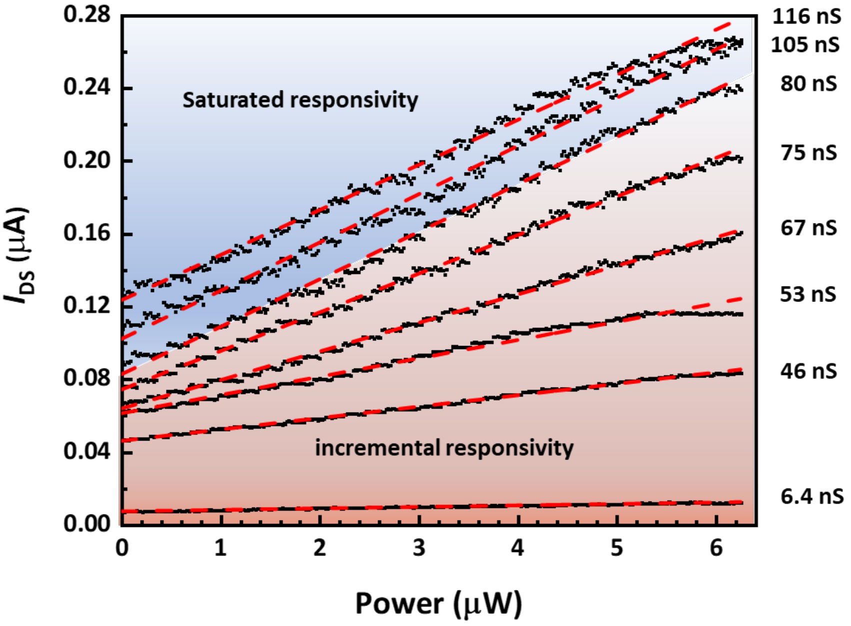

Supplementary Fig. 23 | PMC device2 for repeated validation of responsivity stability at different wavelengths for different conductance states at 1310 nm laser. The dots are the actual measurements and the dashed line is the linear fit curve.

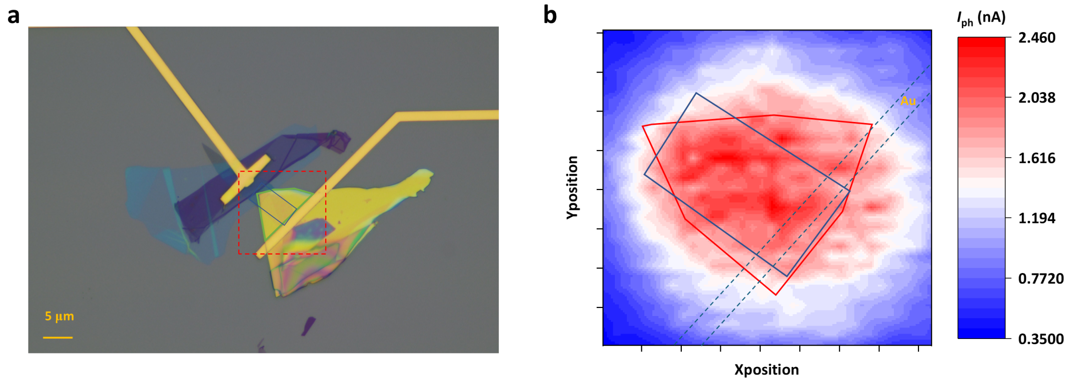

Supplementary Fig. 24 | Photocurrent mapping at 1550 nm laser. a, Image of the device under a 50x optical microscope, scale 5 μm. The red dotted box represents the area used for photocurrent scanning. **b,** Photocurrent mapping at 1550 nm. The solid blue box represents the location of the MoS_2. The solid red box represents the location of BP. The blue dashed box represents the position of the metal electrode. The right panel is the net photocurrent intensity spectrum.

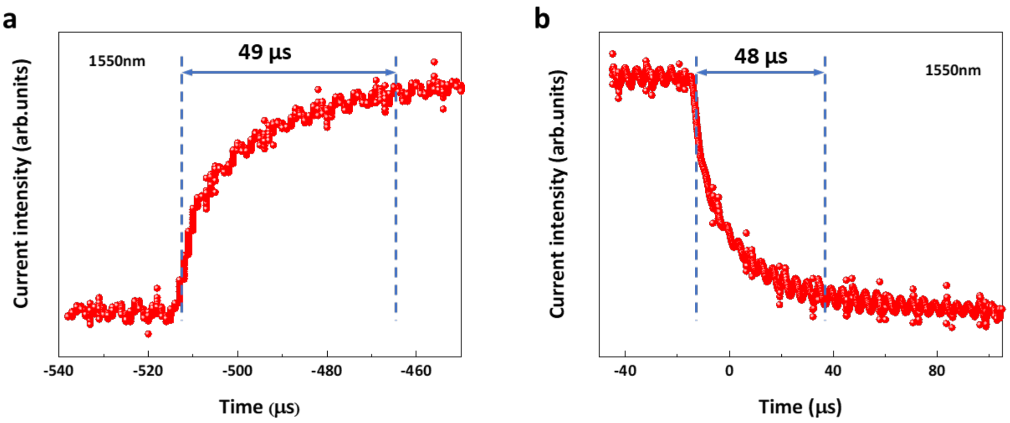

Supplementary Fig. 25 | Response time of the PMC device under 1550 nm laser illumination at V_{DS} = 1 V. a,b, Corresponding τ_{rising} and $\tau_{falling}$ are 49 μs and 48 μs, respectively. τ_{rising} and $\tau_{falling}$ represent the rising time and falling time, respectively

In the near-infrared region, the photon energy is sufficiently high to directly excite electron-hole pairs, generating a photocurrent. Therefore, the photovoltaic effect predominantly governs this region. This direct photo-generated carrier generation process is linear. In the mid-wave infrared region, the photon energy is lower and more susceptible to thermal effects, often producing photo-thermoelectric and photo-bolometric effects[2-4]. When photons are absorbed and converted into thermal energy, a local or overall temperature increase occurs in the material, leading to changes in the photocurrent due to temperature variation. This process is influenced by the duration of illumination, introducing non-linear characteristics. The solution is to get the mapping function by using a fitting method. In **Supplementary Fig. 26**, The red dots are the data from the test, and the red line is an exponential fit to the test data. R^2 = 0.99013, 0.94944, 0.98469 and 0.98597, respectively.

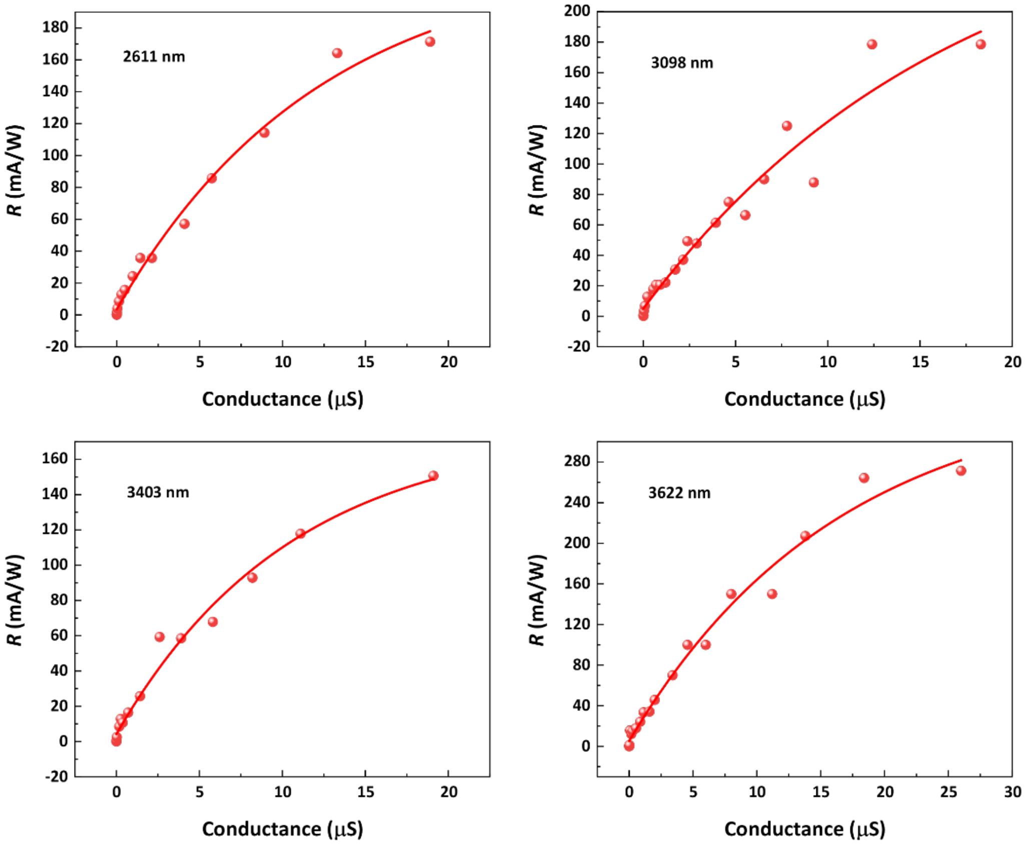

Supplementary Fig. 26 | The photoresponse of the PMC device3 at various conductance states under mid-wave infrared laser stimulation. a, b, c, d, The responsivity increases with the increasing conductance state under 2611 nm, 3098 nm, 3403 nm, and 3622 nm, respectively. The red dots are the data from the test, and the red line is an exponential fit ($y = A\ exp(-x/t) + B$) to the test data. R^2 = 0.99013, 0.94944, 0.98469 and 0.98597, respectively.

Supplementary Note 5. Black radiation response of the PMC device.

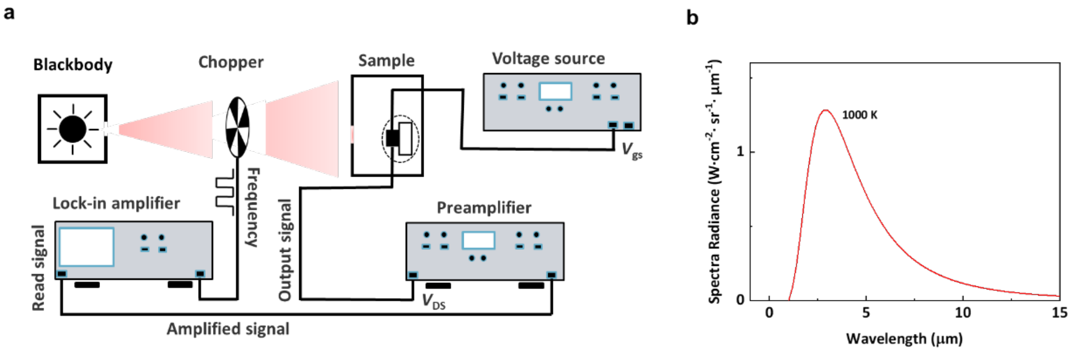

Supplementary Fig. 27 | Blackbody characterization of PMC devices. a, Characterization principle diagram of the blackbody test system. **b,** Relationship between wavelength and radiant power at 1000 K blackbody temperatures.

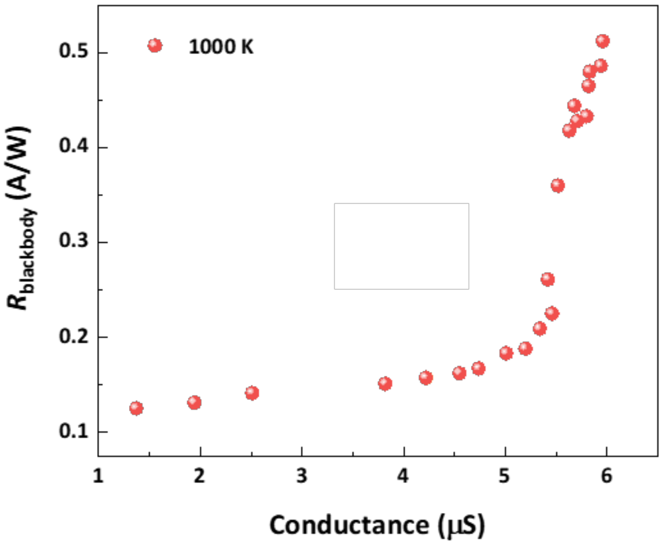

Supplementary Fig. 28 | Blackbody responsivity of PMC devices at various conductance states.

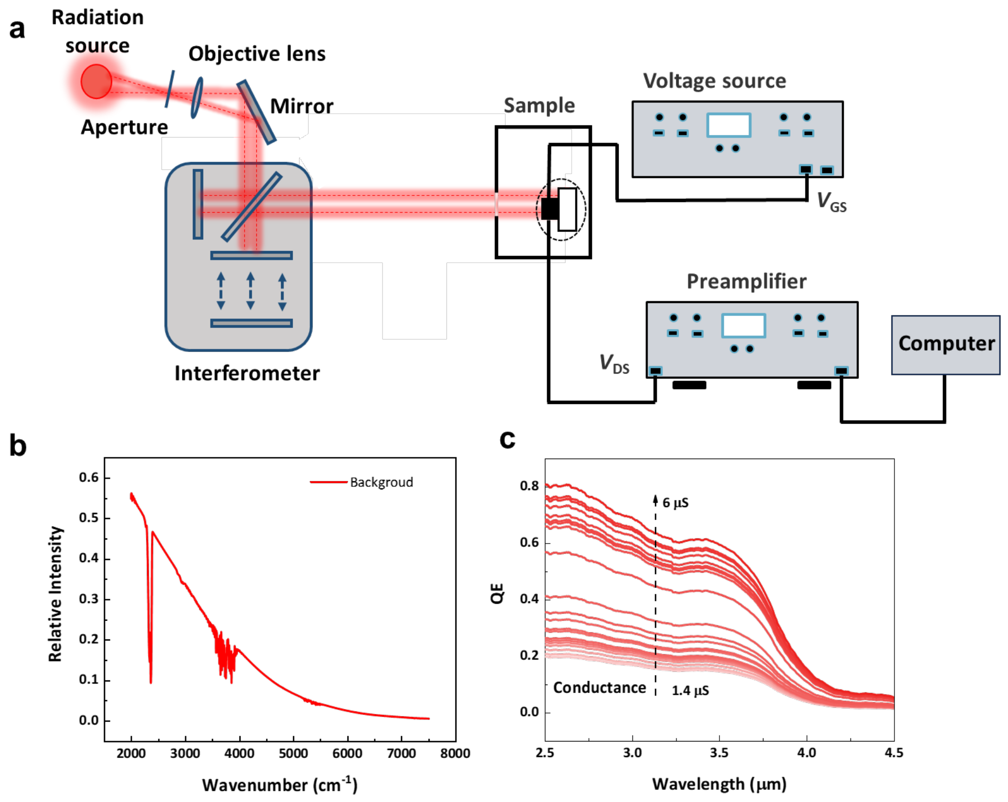

Supplementary Fig. 29 | Fourier transform infrared spectroscopy characterization of PMC devices a, the schematic illustration of the Fourier transform infrared spectrometer. b, Relative response spectrum of the background obtained by the internal (DTGS) detector. **c,** Quantum efficiency (QE) as a function of wavelength at various conductance states. A monochromatic radiation source facilitates spectral response testing, with variations in the radiation source's wavelength yielding the photodetector's response spectrum. Fourier inverse transform is applied, leveraging the time domain characteristics of the sampled signal, to extract the desired spectral response information.

We rebuilt a batch of devices (PMC4) and tested the noise and blackbody response in multiple conductance states (36.8 nS to 3.65 μS) in **Supplementary Fig. 30a b**, respectively. Further, we calculated the detectivity of different conductance states in the mid-infrared (3.5 μm) in **Supplementary Fig. 30c**. The test results indicate that the detectivity (10^9 Jones) of the device remains constant when the conductance state changes.

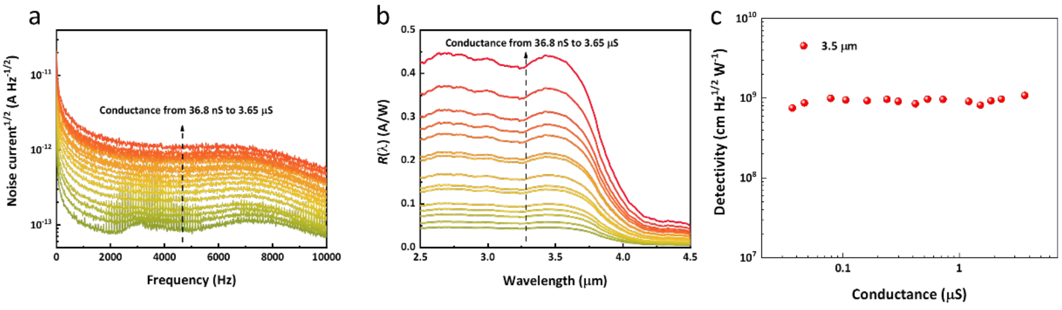

Supplementary Fig. 30 | Mid-infrared detectivity of the PMC device. a, Noise current spectral density in multiple conductance states. **b,** Responsivity as a function of wavelength at various conductance states. Conductance state change from 36.8 nS to 3.65 μS at V_{DS} = 0.1 V. **c,** Specific detectivity of different conductance states in the mid-infrared (3.5 μm).

Calculations of blackbody detection

The total incident power of blackbody radiation on the device surface can be calculated using the formula[5]:

(1)

Where α is the modulation factor, ε is the average emissivity of the blackbody radiation source, σ is the Stefan-Boltzmann constant. T is the blackbody radiation source temperature, T_0 is the test environment temperature, A is the blackbody radiation source area, A_n is the device area and L is the distance of the photodetector from the aperture.

The equation of responsivity (   ) under blackbody radiation is:

(2)

where I_{ph} is blackbody photocurrent.

The response spectrum obtained by FTIR is only a relative response spectrum. The response spectrum measured by FTIR is an equal power curve in which the radiated power at any wavelength is equal. After calibration of the blackbody response, the responsivity of the response spectrum of the photodetector can be obtained. The relative responsivity spectrum $R'(\lambda)$ is obtained from the FTIR characterization:

(3)

Since the blackbody radiation has a continuous spectrum and the emissivity of each wavelength is different, the blackbody light signal generated by the photodetector is the sum of the signals generated by the radiation at each wavelength:

(4)

The ratio of the blackbody responsivity ($R_{\text{blackbody}}$) to the peak responsivity ($R(\lambda_p)$) of an infrared photodetector is a constant, the g-factor. The g-factor can be calculated from the tested $R'(\lambda)$ and the blackbody spectral emissivity ($\phi(\lambda)$). Therefore, by calculating the g-factor, the peak responsivity ($R(\lambda_p)$), quantum efficiency ($QE(\lambda_p)$) and the peak detectivity ($D^*(\lambda_p)$) can be obtained:

or (5)

(6)

(7)

where h is Planck's constant, c is the speed of light in vacuum, λ is the photon wavelength, is the electrical bandwidth.

Supplementary Note 6. Image processing application of the PMC device.

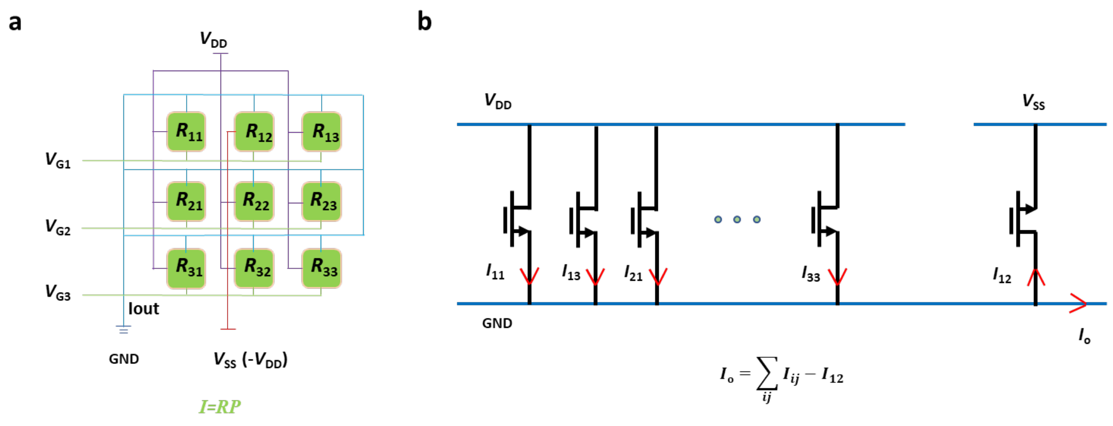

Supplementary Fig. 31 | Circuit connection to realize negative weight. a, Taking the 3×3 for example, by introducing V_{SS} ($-V_{DD}$) to realize the opposite current path. **b,** Equivalent circuit diagram. Leveraging Kirchhoff's law enables the implementation of current subtraction, representing the incorporation of negative weights in the convolution kernel.

Morphological image processing contains erosion and dilation[6]. Erosion operation decreases the gray value, leading to an overall reduction in the brightness of the output image after the erosion compared to the original image. This operation makes the areas in the image smaller and the darker regions larger. Conversely, dilation is similar to an erosion operation, where brighter objects in the image become enlarged in the image while reducing the size of darker objects.

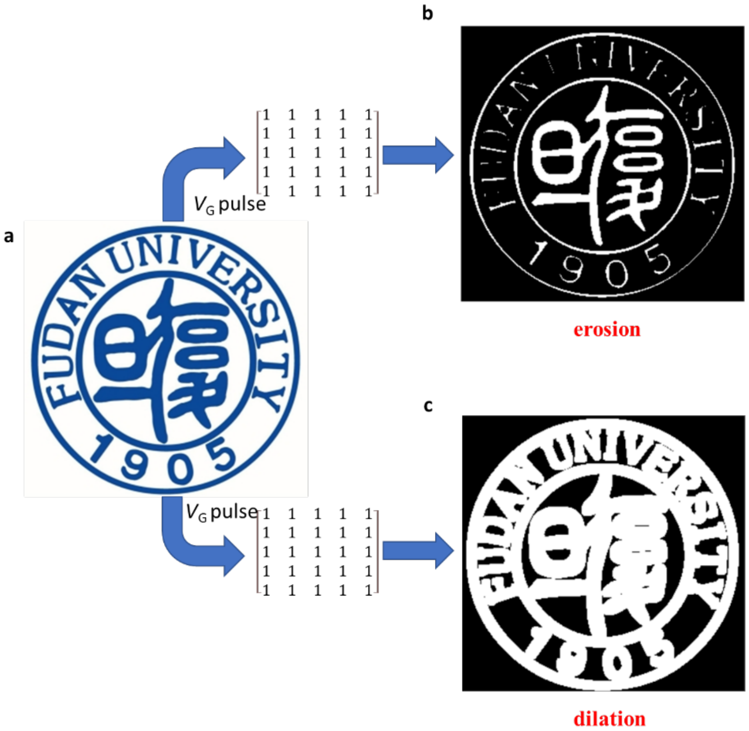

Supplementary Fig. 32 | morphological image processing (erosion and dilation). a, School badge of Fudan University. **b,** Image after erosion operation. **c,** Image after dilation operation.

The edge of an image represents its fundamental feature, where "edge" refers to the local characteristic discontinuity in the image. Mutations in grayscale or structural information are identified as edges, including variations in gray level, the mutation of color, the mutation of texture structure and so on. An edge makes the transition from one region and to another, and leveraging this characteristic facilitates image segmentation. Edge detection is usually carried out by Sobel, Scharr and Laplacian operators, which can be replaced by device weights[7].

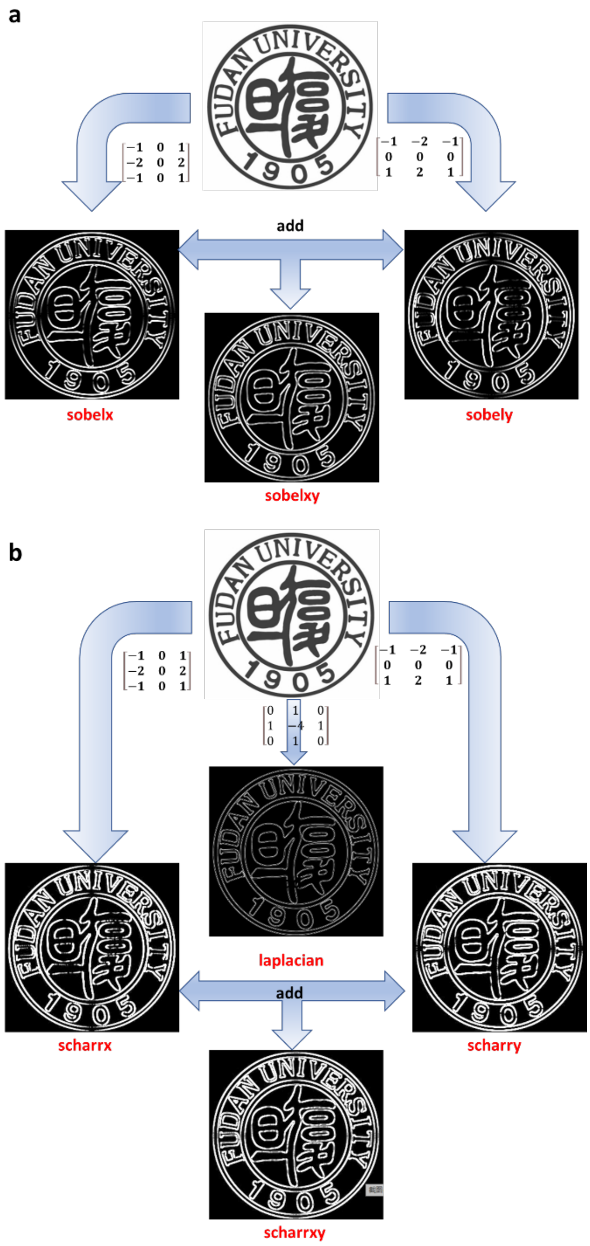

Supplementary Fig. 33 | Edge detection operation (Sobel, Scharr and Laplacian). **a,** Sobel operation and corresponding kernels. **b,** Scharr, Laplacian operation and corresponding kernels.

Faster-RCNN networks

Faster-RCNN, a pivotal two-stage object detection algorithm, starts by locating anchor rectangular boxes in images, involving binary classification of background and target objects[8]. The subsequent stage classifies the objects to be detected in the anchor boxes. This architecture comprises four primary parts: a, Convolution (Conv) layers. Using basic conv+relu+pooling layers, Faster RCNN extracts feature maps, shared across subsequent layers, including the Region Proposal Network (RPN) and fully connected layers. b, Region Proposal Networks. RPN network is used to generate region proposals,employing softmax to classify anchors as positive or negative. Bounding box regression adjusts anchors to obtain accurate proposals. c, Roi Pooling. This layer gathers the input feature maps and proposals, extracting proposal feature maps set to subsequent fully connected layer for determining the target category. d, Classification. Proposal feature maps are used to calculate the category, followed by bounding box regression to pinpoint the precise position of the detection box.

The training of Faster R-CNN involves continuing to train on a model that has already been trained (ResNet50). Train the RPN network on the pre-trained model. The two stages of Faster RCNN, RPN and the subsequent classification network, both output coordinate regression values and classification values, so the loss functions of both networks can be expressed using the following formula[9]:

(7)

Where is explaining the anchor index. positive softmax probability. The

symbol represents the probability of the corresponding GT(ground truth) prediction. L_{reg} is the loss of rpn_loss_anchor layer, L_{cls} is the loss of rpn_classification_loss layer. N_{cls} is the number of network trainings used for anchor classification, N_{reg} is the number of network trainings used for bounding box regression.

The concept of IOU is a measure of how much the predicted frame overlaps with the true frame, which is meant to elicit the other two indicators: precision and recall.

(8)

Where $S_{Intersection}$ represents the overlapping area between the predicted and actual boxes, S_{Sum} corresponds to the total area occupied by both the prediction and actual boxes.TP (True Positives) means that they are divided into positive samples and they are divided correctly. TN (True Negatives) means that the sample was divided into negative samples, and it was divided correctly. FP (False Positives) means that the sample was divided into positive samples, but was divided into wrong samples (in fact, the sample was negative). FN (False Negatives) means that the sample was divided into negative samples, but the division was wrong (in fact, the sample was positive). Furthermore, there are two important concepts:

(9)

(10)

Average Precision (AP) in fact refers to the area under the curve drawn using

different combinations of Precision and Recall's points. By considering different confidence levels, diverse Precisions and Recalls are obtained. With sufficiently dense confidence levels, numerous Precisions and Recalls can be acquired.. Precision and Recall can form a curve, and the area beneath this curve represents the AP value for a specific class. The mean Average Precision (mAP) is the average of the AP values across all classes, providing a comprehensively evaluation of recognition results.

Supplementary Table 2. Response weight sequence (A/W).

0.41517	0.4351	0.46831	0.50152	0.52145	0.53806	0.55466
0.60781	0.62441	0.69416	0.7473	0.86687	1.19568	1.38832
1.42153	1.43814	1.47468	1.54442	1.59424	1.61417	1.70053

Supplementary Table 3 Trained weight.

0.02831	0.01813	0.0159	0.00289	-0.05268	-0.04726	0.01888
0.00816	0.02476	0.07657	0.09053	-0.01876	-0.07937	-0.01499
-0.02725	-0.06584	0.01985	0.18494	0.11569	-0.08993	-0.09427
-0.00957	-0.07922	-0.12999	0.09536	0.27019	0.09944	-0.0146
0.02329	-0.04632	-0.1831	-0.15598	0.05369	0.08355	0.03727
0.04218	0.04595	-0.04669	-0.12698	-0.02087	0.03328	0.02522
0.01452	0.0351	0.00392	-0.06041	-0.01689	0.01182	0.00491

Supplementary Table 4 PMC device weight.

0.02729	0.02056	0.01384	0	-0.05216	-0.0488	0.02056
0	0.02729	0.07704	0.09053	-0.02056	-0.07704	-0.01384
-0.02729	-0.06628	0.02056	0.1678	0.10124	-0.10124	-0.10124
0	-0.07704	-0.10124	0.10124	0.27	0.10124	-0.01384
0.02056	-0.0488	-0.1678	-0.1678	0.05216	0.07704	0.03804
0.03804	0.0488	-0.0488	-0.10124	-0.02056	0.03468	0.02729
0.01384	0.03468	0	-0.06628	-0.02056	0.00981	0

Supplementary Table 5 Comparison between the PMC image processing system and commercial uncooled MCT MIR conventional image processing system.

	Sensor					ADC conversion	Memory write/ frame	Processor load image / frame	Process of reasoning	Programming time / energy	System time / energy
	Response	Device time	Effective area/pixel	Cost	Substrate						
PMC image processing system	1.68A/W	49 µs	~100 µm^2	Cheap	SiO_2/Si	\	\	\	Within the sensing process	~200 µs / ~180nJ	>249 µs / >180nJ
Conventional	~1A/W	120 ns	1 mm^2	Expensive ￥12503 / pixel	CdZnTe	10-50 µs / ~60mW	0.2 ms / 3.3W	10 µs / ~5W	100 ms/ ~6W	\	>100 ms/ >600mJ

* Take the processing of a 1MB image as an example

* The data of commercial uncooled MCT detector used in conventional image processing system comes from VL5T0 (THORLABS)

* The memory in conventional image sensors uses data from Solid-State Disk (SSD)

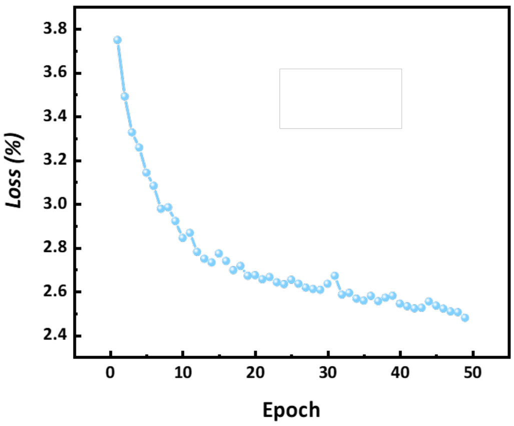

Supplementary Fig. 34 | Losses during training. Over the first 50 epochs, the training losses show a convergent trend.

Supplementary References

1. Liu, L. et al. Ultrafast non-volatile flash memory based on van der Waals heterostructures. *Nat. Nanotechnol.* **16,** 874-881 (2021).
2. Low, T., Engel, M., Steiner, M. & Avouris, P. Origin of photoresponse in black phosphorus phototransistors. *Phys. Rev. B* **90,** (2014).
3. Deng, Y. et al. Black Phosphorus–Monolayer MoS2 van der Waals Heterojunction p–n Diode. *ACS Nano* **8,** 8292-8299 (2014).
4. Youngblood, N., Chen, C., Koester, S. J. & Li, M. Waveguide-integrated black phosphorus photodetector with high responsivity and low dark current. *Nat. Photon.* **9,** 247-252 (2015).
5. Wang, F., Zhang, T., Xie, R., Wang, Z. & Hu, W. How to characterize figures of merit of two-dimensional photodetectors. *Nat. Commun.* **14,** 2224 (2023).
6. Wang, C.-Y. et al. Gate-tunable van der Waals heterostructure for reconfigurable neural network vision sensor. *Sci. Adv.* **6,** eaba6173 (2020).
7. Vincent, O. R. & Folorunso, O. A descriptive algorithm for sobel image edge detection. *Proceedings of informing science & IT education conference (InSITE)*; 2009; 2009. p. 97-107.
8. Sun, X., Wu, P. & Hoi, S. C. Face detection using deep learning: An improved faster RCNN approach. *Neurocomputing* **299,** 42-50 (2018).
9. Ren, S., He, K., Girshick, R. & Sun, J. Faster r-cnn: Towards real-time object detection with region proposal networks. *Advances in neural information processing systems* **28,** (2015).
